# Neuroendoscopy and Postoperative Nausea and Vomiting: Pathophysiology, Incidence and Management Strategies

**DOI:** 10.3390/brainsci15060586

**Published:** 2025-05-29

**Authors:** Vincenzo Pota, Francesco Coletta, Francesca Pascazio, Pasquale Rinaldi, Antonio Tomasello, Giovanna Paola De Marco, Francesca Schettino, Maria Beatrice Passavanti, Pasquale Sansone, Maria Caterina Pace, Manlio Barbarisi, Roberto Altieri, Romolo Villani, Francesco Coppolino

**Affiliations:** 1 Department of Women, Child, General and Specialistic Surgery, University of Campania «L. Vanvitelli», 80138 Naples, Italy; vincenzo.pota@unicampania.it (V.P.); francesca.pascazio@studenti.unicampania.it (F.P.); mariabeatrice.passavanti@unicampania.it (M.B.P.); pasquale.sansone@unicampania.it (P.S.); mariacaterina.pace@unicampania.it (M.C.P.); francesco.coppolino@unicampania.it (F.C.); 2 Emergency Anesthesia, Burn Intensive Care Unit and Poison Control Center, AORN Antonio Cardarelli, 80131 Naples, Italy; pasquale.rinaldi1994@gmail.com (P.R.); antonio.tomasello@aocardarelli.it (A.T.); giampaolademarco29@gmail.com (G.P.D.M.); francesca.schettino@aocardarelli.it (F.S.); romolo.villani@aocardarelli.it (R.V.); 3 Multidisciplinary Department of Medical-Surgical and Dental Specialities, University of Campania «L. Vanvitelli», 80138 Naples, Italy; manlio.barbarisi@unicampania.it (M.B.); roberto.altieri@unicampania.it (R.A.)

**Keywords:** neuroendoscopy, PONV, craniotomy, antiemetics, risk factors, minimally invasive neurosurgery

## Abstract

Neuroendoscopy is a minimally invasive surgical technique used to treat brain pathologies such as hydrocephalus, arachnoid cysts, and skull base tumors. While it offers several advantages, including reduced tissue trauma and lower morbidity, it is associated with a high risk of postoperative nausea and vomiting (PONV). This paper provides a narrative review of the literature on the incidence, pathophysiology, and management of PONV in patients undergoing neuroendoscopic procedures. The review includes several studies published between 2001 and 2024, analyzing specific risk factors such as female gender, postoperative opioid use, extended endoscopic approaches, and cavernous sinus dissection. PONV prevention strategies include a multimodal approach combining total intravenous anesthesia (TIVA) with propofol, perioperative hydration, and pharmacological prophylaxis (5-HT3 receptor antagonists, NK1 antagonists, dexamethasone, and droperidol). Despite advances in surgical and anesthetic techniques, further research is needed to develop procedure-specific protocols and optimize PONV management in neuroendoscopy.

## 1. Introduction

Cranial endoscopy is a minimally invasive technique that employs an endoscope to access target sites through natural cavities or predefined pathways. It is indicated for pathologies causing cerebrospinal fluid pathway obstructions, such as hydrocephalus, arachnoid cysts, various intraventricular lesions, hypothalamic hamartomas, craniosynostosis, skull base tumors, and spinal lesions. Techniques to restore cerebrospinal fluid circulation include third ventriculostomy, septostomy, foraminoplasty, aqueductoplasty, stent placement, and the excision of cysts and tumors [1].

Neuroendoscopic surgery is widely employed whenever feasible, as it minimizes the incidence of complications and ensures favorable clinical outcomes. By utilizing an endoscope and specialized instruments, neurosurgeons can perform complex procedures through small incisions, a key advantage for invasive brain surgeries [2]. In endoscope-assisted microsurgery, procedures are predominantly performed under microscopic visualization, ensuring high-resolution imaging. The endoscope is selectively deployed at specific stages to enhance visualization of anatomical regions obscured by bony or dural structures, as well as neurovascular components. This approach reduces the need for extensive tissue retraction or cranial base perforation, thereby improving surgical precision and safety [3].

The use of an endoscope in brain surgery allows the neurosurgeon to achieve optimal illumination and visualization of the surgical target, even in cases where the operative field is particularly deep. Additionally, the endoscope provides a significant advantage due to its extensive depth of field, enhancing the surgeon’s ability to navigate the surgical area [4]. From an anesthetic perspective, the management of neuroendoscopic procedures must account for the high risk of postoperative nausea and vomiting (PONV). The occurrence of PONV is not only an uncomfortable experience for patients but also a significant concern due to its potential to cause serious complications, including aspiration, pneumonia, dehydration, electrolyte imbalances, and metabolic alkalosis. Moreover, the combination of vomiting and pain can elevate arterial blood pressure and intracranial pressure, increasing the risk of intracranial hemorrhage. PONV can also prolong hospital stays, thereby increasing healthcare costs and negatively impacting patient satisfaction [5,6]. The risk of PONV in neurosurgery may be related to the proximity of neural centers controlling vomiting to the operative area [7].

Notably, a meta-analysis of 17 clinical trials has provided valuable insight into optimal prophylactic strategies for PONV. The results indicate that ramosetron appears to be the most effective prophylactic agent for preventing PONV within 24 h of craniotomy, with higher rates of complete response. Fosaprepitant was identified as the most effective option for preventing postoperative vomiting (POV) in the first 0–24 and 0–48 h. Both agents may be even more effective when used in combination with perioperative dexamethasone. These results may guide clinicians toward more effective pharmacologic prophylaxis with fewer adverse effects, ultimately improving patient outcomes.

While much of the existing literature focuses on traditional craniotomy with a neurosurgical approach, this review also seeks to establish a framework for the management of PONV in neuroendoscopic procedures, a more recent and innovative technique. This dual perspective is essential to optimize perioperative care across the spectrum of modern neurosurgical procedures.

## 2. Materials and Methods

We conducted a narrative review on the incidence of postoperative vomiting in brain endoscopic surgeries, which we believe is crucial to provide a comprehensive perspective on this topic in response to emerging innovative surgical techniques.

Using the PubMed—Medline database and Google Scholar, we scrutinized all pertinent papers (narrative reviews, systematic reviews and meta-analyses, randomized controlled trials, prospective studies, case reports) using selected keywords: neuroendoscopy, cerebrum, cerebral, brain, postoperative nausea and vomiting, postoperative, nausea, vomiting, PONV.

The timeline of the studies included ranged from 2001 to 2024. A total of 54 articles were selected, with the following inclusion criteria: meta-analyses, narrative or systematic reviews, RCTs, prospective studies, retrospective studies, case-control studies, case reports, and case series (Table 1).

This article relies on previously published studies and does not present any original research involving human or animal subjects conducted by the authors.

## 3. Pathophysiology of PONV and Risk Factors Technique Related

The neuroanatomical region responsible for the regulation of nausea and vomiting is a poorly defined area referred to as the “vomiting center,” located within the lateral reticular formation of the brainstem. This center integrates afferent inputs from various sources, including higher cortical regions, the cerebellum, the vestibular apparatus, and the vagus and glossopharyngeal nerves. Additionally, it interacts with the nucleus tractus solitarius and the chemoreceptor trigger zone (CTZ), the latter being situated in the floor of the fourth ventricle [17].

The neurotransmitters involved in controlling nausea and vomiting include the following:

- Acetylcholine (muscarinic receptors);
- Dopamine (D2 receptors);
- Histamine (acting on H1 receptors);
- Substance P (activating the NK-1 receptor);
- Serotonin (activating the 5-HT3 receptors);
- Cannabinoids (activating the CB1 receptors).

Postoperative nausea and vomiting (PONV) can extend recovery room time, increase hospital stay, and negatively impact the patient’s mental well-being. Several factors have been identified as being associated with an increased risk of PONV. These include female gender, non-smoking status, a history of motion sickness and PONV in previous surgical procedures, younger age, comorbidities such as migraine and obesity, use of inhalational agents during anesthesia, including nitrous oxide (N_2_O), prolonged anesthesia duration, type of surgical procedure and the presence of postoperative pain [17].

Given the negative impact of PONV on patient outcomes, it is crucial to identify surgical procedures with a higher likelihood of being associated with PONV. For instance, the reported incidence of PONV following craniotomy ranges between 22% and 70% in the absence of prophylactic measures [10,18]. In recent years, neurosurgical teams specialized in skull base surgery have increasingly favored the endoscopic endonasal approach. This preference is due to its association with reduced perioperative morbidity, while still maintaining comparable surgical outcomes and low mortality rates. Notably, at academic medical centers, up to 20% of all primary brain tumors are now managed using an endoscopic approach [19,20].

The simplified Apfel score is a widely used objective method for assessing the risk of PONV incidence after a surgical procedure. This scoring system identifies four PONV-related factors as key risk predictors: female gender, non-smoking status, a history of PONV, and the use of postoperative opioids (Table 2: Apfel Score) [15].

A multimodal strategy could provide an optimal balance between effective pain management and medication-related adverse effects, such as postoperative nausea and vomiting, which should be prevented, as previously stated, to minimize the risk of elevated intracranial pressure [21]. Total intravenous anesthesia (TIVA), especially with propofol, is particularly well-suited for neuroanesthesia, where it is recommended to facilitate brain relaxation during surgery [22].

Proper hydration and systematic perioperative oxygen therapy may reduce the risk of postoperative nausea and vomiting. In a study involving 200 patients, pre-induction hydration with 20 mL/kg of a crystalloid solution reduced PONV by 50% in the first 24 h after surgery. Others propose a multimodal approach combining various measures, including hydration, pharmacological prophylaxis, and total intravenous anesthesia without nitrous oxide [23].

Although numerous risk factors for PONV have been identified, including those related to anesthesia, surgical procedures, and individual patient characteristics, and while currently available data suggest an association between neuroendoscopic procedures and PONV, there remains a lack of specific data regarding the risk factors for PONV in endoscopic skull base surgery [16,24].

A retrospective study published by Sato et al. [9]. suggested that spontaneous intracranial hypotension may be responsible for an increased incidence of nausea and vomiting. This suggests that a reduction in cerebrospinal fluid (CSF) may influence the incidence of PONV following various types of craniotomy. Supporting this hypothesis, cerebrovascular and transsphenoidal surgery involving complicated CSF leaks (all procedures associated with a greater volume of CSF removal) have been correlated with a higher incidence of postoperative nausea and vomiting (PONV) [9,25].

Endoscopic skull base surgery is considered a high-risk surgical technique for PONV. Alongside patient-related PONV risk factors, it is crucial to consider factors related to the surgical technique itself, including the site and proximity to the vomiting centers in the nervous system. A retrospective review conducted between July 2018 and August 2019 examined data from 99 patients who underwent endoscopic skull base surgery. The incidence of vomiting significantly increased in cases involving cavernous sinus dissection, female patients, and surgeries with an extended surgical approach. No correlation was found between nausea and vomiting episodes and the total duration of surgery or the opioid dose administered postoperatively [7].

The literature highlights predictive factors for PONV in neuroendoscopic procedures. It also suggests that antiemetic therapy should always be considered in the presence of predictive factors such as female gender, cavernous sinus dissection, and extended surgical approaches [7,15].

## 4. Strategies and Medications for the Prevention of Postoperative Nausea and Vomiting (PONV)

Beyond the available medications, the choice of anesthetic management and the type of drugs used play a crucial role in the prevention of PONV.

Among the most documented and effective antiemetic drugs currently used for PONV prophylaxis in neuroendoscopy are “setrons” (5-HT3 receptor antagonists), NK1 antagonists (aprepitant), glucocorticoids such as dexamethasone, and dopaminergic agents like droperidol (D2 antagonists) [16].

Furthermore, as previously mentioned, the choice of anesthetic drugs used can significantly influence the incidence of postoperative nausea and vomiting (PONV). Dexmedetomidine, a selective α-2 agonist, has been used as an adjunct analgesic during and after neuroendoscopic procedures, and it has been linked to a decrease in the incidence of PONV [11,26]. In a randomized controlled trial (RCT) including 80 patients undergoing sevoflurane-fentanyl anesthesia, Peng et al. found that adding dexmedetomidine infusion resulted in fewer events requiring rescue medication for PONV within the first 90 min after surgery compared to the placebo group (*p* = 0.005) [11]. Another RCT by Gupta et al., involving 50 patients undergoing supratentorial surgery with general anesthesia and intraoperative dexmedetomidine or fentanyl infusion, reported a PONV incidence of 8% and 0%, respectively, in the fentanyl and dexmedetomidine groups [26].

Additionally, the addition of scalp blocks to the anesthetic technique in both supratentorial and infratentorial surgery reduces pain, leading to less opioid consumption and a lower incidence of PONV up to 72 h postoperatively [12].

In a recent study, Chen, Xi et al. [8] also evaluated the efficacy of intraoperative gastric tube placement in preventing PONV during transnasal neuroendoscopic procedures. The results demonstrated a significant reduction in nausea in the gastric tube group compared to the control group (*p* < 0.01), while the frequency of vomiting, although lower, did not reach statistical significance (*p* > 0.05) [8].

Below, we specifically analyze the potential pharmacological strategies for the prevention of postoperative nausea and vomiting (Table 3).

### 4.1. 5-HT3 Serotonin Receptor Antagonists (5-HT3 ARs)

The available preparations include ondansetron, dolasetron, granisetron, and tropisetron. Only ondansetron is approved for specific use in PONV. It acts both centrally and peripherally. Ondansetron acts centrally as receptor antagonists of serotonin 5-HT3 receptors located in the area postrema, while peripheral effects are mediated by action on 5-HT3 receptors located at the vagus nerve terminals. A dose of 4 mg is effective [27].

In a study involving 40 patients undergoing subtentorial neuroendoscopic surgery, ondansetron significantly reduced PONV in the first 48 h (15% versus 65% in the placebo group) and the use of rescue antiemetic (10% versus 45% in the placebo group). Drowsiness was noted in 30% of patients receiving ondansetron [28]. In another study, 60 patients undergoing supratentorial surgery were administered either 4 mg of ondansetron, 0.625 mg of Droperidol, or a placebo. The incidence of nausea was reduced in both active groups, but only Droperidol was effective against vomiting. At the doses used, Droperidol was no more sedative than ondansetron [29].

In a subsequent study, the same team evaluated 8 mg of ondansetron versus a placebo in 50 patients undergoing subtentorial surgery. Following ondansetron, the risk of nausea was reduced by one-third in the first 12 postoperative hours (*p* = 0.036). The vomiting frequency in the first 24 h decreased from 61% after placebo to 26% after ondansetron (*p* = 0.036). Pain and sedation scores were identical in both groups [30]. Lastly, the largest study involved 152 patients undergoing supratentorial and subtentorial neurosurgery, receiving either 4 mg of ondansetron or a placebo. Ondansetron reduced the frequency of vomiting in the first 24 h from 39% to 11% (*p* = 0.01). However, the reduction in nausea was not statistically significant [31].

The use of ondansetron could be associated with side effects. Among the most common (occurring in over 10% of adult patients) are headaches, fatigue, dry mouth, malaise and constipation. Less frequently, central nervous system manifestations such as sedation and drowsiness, as well as local reactions at the injection site and pruritus, are observed. A temporary increase in liver function test values has also been noted. Additionally, the use of ondansetron is associated with the risk of serotonin syndrome, with this risk being notably higher when ondansetron is administered in combination with other serotonergic drugs [32,33]. In addition to the aforementioned side effects, although of limited clinical significance, ondansetron administration may lead to changes in the ECG, such as QTc prolongation. These changes occur within 1–2 h after administration and resolve within 24 h. Nonetheless, the primary concern is the potential risk of torsades de pointes and other arrhythmias, similar to other drug categories that cause QTc prolongation. The highest risk for such complications is associated with intravenous administration. Due to this risk, the FDA does not recommend single doses exceeding 16 mg intravenously [34].

Ondansetron is contraindicated in patients with known hypersensitivity to the drug or any of its components. Additionaly, it should not be administred to patients receiving apomorphine, as their combination may result in severe hypotension and loss of consciousness. Caution is required in patients with phenylketonuria (PKU), as certain tablet formulations may contain phenylalanine. Classified as a category B drug for pregnacy risk, ondansetron should only be used when other treatments have proven ineffective in managing nausea and vomiting associated with pregnancy or hyperemesis gravidarum. Before considering ondansetron, alternative therapeutic options such as antihistamines (Diphenhydramine and Meclizine) or dopamine antagonists (Metoclopramide and Promethazine) should be evaluated in this patient population [35].

### 4.2. Droperidol

Droperidol is a long-acting butyrophenone with a potent antiemetic effect that can last for 8 h or more. Its mechanism of action involves interactions with multiple receptor classes, primarily exerting an antagonistic effect on dopaminergic receptors, with lesser activity on adrenergic, serotonergic and GABAergic receptors. Like other drugs that act on these receptor classes, droperidol is associated with various side effects. The most common adverse reactions include sedation, agitation (particularly in pediatric patients), dysphoria and dyskinesia. For this reason, its use is discouraged in patients with Parkinsonism or other conditions involving central dopamine depletion. The standard dose is approximately 75 mcg/kg, equivalent to around 5 mg for a 70 kg adult. However, at these doses, side effects are frequent, particularly sedation, which may prolong the recovery phase after anesthesia [36].

To mitigate these adverse effects, some clinical controlled studies have evaluated the efficacy of lower doses. Among the different strategies, a dose of 5 mcg/kg may be beneficial, though it does not always provide sufficient efficacy, especially in high-risk surgical procedures or in more vulnerable patients. A dose of 10 mcg/kg is considered more reliable but often requires additional administration during the recovery phase. In cases of high postoperative nausea and vomiting (PONV) risk, a dose of 20 mcg/kg seems to offer the best balance between therapeutic effectiveness and minimal sedative or agitation effects [37].

### 4.3. Dexamethasone

Dexamethasone, at doses of 3 to 12 mg, especially when combined with 5-HT3 antagonists, is frequently used in PONV management [38]. The potential mechanisms of dexamethasone are related to its anti-inflammatory effect mediated by the antagonism of prostaglandins and its direct action on the solitary tract nucleus [39]. Furthermore, in surgical patients, there is substantial evidence that dexamethasone can reduce pain and thereby potentially reduce opioid consumption, which may, in turn, decrease the nausea and vomiting associated with opioids [40].

Overall, the evidence supports the prophylactic administration of dexamethasone in surgical patients when necessary, at the time of anesthesia induction. This reflects the perceived inability of dexamethasone to develop a rapid antiemetic effect and, unlike classic antiemetics that block receptor systems, the antiemetic mechanisms of dexamethasone may be indirect and thus delayed [41]. Among its side effects, a postoperative increase in blood glucose levels has been noted [42].

### 4.4. Metoclopramide

Metoclopramide has been approved by the FDA for the treatment of postoperative nausea and vomiting, particularly in patients with gastroesophageal reflux disease or diabetic gastroparesis [43]. Its mechanism of action is based on dopamine receptor antagonism at both central and peripheral levels within the chemoreceptor trigger zone in the medulla, specifically in the area postrema, a region typically stimulated by levodopa or apomorphine. This effect is achieved by reducing the sensitivity of afferent visceral nerve fibers that transmit signals from the gastrointestinal tract to the vomiting center in the area postrema [44].

Additionally, metoclopramide counteracts the antiperistaltic effects of apomorphine, promoting gastric emptying and enhancing the amplitude and duration of esophageal contractions. Furthermore, it increases the basal tone of the lower esophageal sphincter while relaxing the duodenal bulb and the pyloric sphincter, thereby facilitating the transit of intestinal contents through the duodenum and jejunum [45].

Metoclopramide can be administered orally in the form of tablets or solution, typically at doses ranging from 5 to 10 mg. Alternatively, it can be administered intravenously or intramuscularly in patients with severe nausea for whom oral administration is not an option. When given intravenously, the onset of action is faster even at the same dosage [44]. Adverse effects associated with metoclopramide administration include extrapyramidal symptoms, such as acute dystonic reactions, torticollis, trismus, opisthotonus, akathisia, dystonia, oculogyric crisis, laryngospasm, hyperprolactinemia, tardive dyskinesia, Parkinson’s symptoms, and neuroleptic malignant syndrome. If neuroleptic malignant syndrome occurs following metoclopramide use, the drug must be immediately discontinued, and treatment with dantrolene should be initiated promptly. These adverse effects are related to the drug’s pharmacodynamics, specifically its antidopaminergic mechanism of action. It is important to note that the onset of these symptoms is independent of the dosage and that they are generally reversible upon drug discontinuation [46]. Metoclopramide is contraindicated in patients with the following conditions: gastrointestinal bleeding, intestinal obstruction, intestinal perforation, pheochromocytoma, seizures, depression, Parkinson’s disease, or a history of tardive dyskinesia. The suggested superiority of metoclopramide over ondansetron, as indicated in one study, is an isolated result [47].

### 4.5. Aprepitant

Aprepitant is a drug indicated for the prevention and management of chemotherapy-induced nausea and vomiting (CINV) as well as postoperative nausea and vomiting (PONV) [48].

It functions as a highly selective neurokinin-1 (NK1) receptor antagonist, acting on G-protein-coupled receptors found in both the central and peripheral nervous systems. These receptors primarily bind substance P, a neurotransmitter involved in nociceptive signaling [49]. Centrally, aprepitant is believed to significantly reduce the likelihood of triggering the complex vomiting reflex. Peripherally, NK1 receptors are distributed throughout the gastrointestinal tract, and aprepitant’s interaction with these receptors may dampen vagal afferent signaling, further contributing to its antiemetic properties [50]. For the prevention of anesthesia-induced postoperative nausea and vomiting, aprepitant is administered orally at a dose of 40 mg within three hours prior to anesthesia induction. Given its higher cost compared to other antiemetic agents commonly used in anesthetic practice, its use is recommended primarily for high-risk patients or those in whom vomiting could compromise surgical repair [51].

Aprepitant is generally well tolerated, with minimal side effects, including headache, fatigue, anorexia, constipation, diarrhea, and hiccups [52].

### 4.6. Scopolamine

A promising antiemetic option for reducing the incidence of PONV is the 1.5 mg transdermal scopolamine patch [14]. Due to its pharmacokinetic and pharmacodynamic properties, the patch must be applied several hours before the surgical procedure, with the possibility of placement the night before. It is typically used in combination with ondansetron and Dexamethasone for PONV management. Scopolamine is an alkaloid that competitively inhibits the action of acetylcholine at muscarinic receptors, exerting its effects both centrally and peripherally. Common adverse effects include dry mouth, visual disturbances and sedation. Additionally, as an antimuscarinic agent, scopolamine may cause anticholinergic side effects such as tachycardia, urinary retention and acute angle closure glaucoma [53].

When assessing the effectiveness of transdermal scopolamine in reducing PONV, it is crucial to carefully evaluate its risk/benefit ratio. Adverse effects such as mydriasis and sedation may interfere with postoperative neurological assessment and complicate the detection of potential intracranial hypertension [54,55].

When nausea or vomiting occurs upon discharge from the postoperative recovery room, it is strongly recommended to investigate a contributing medical factor. This could be a reaction to morphine administered for postoperative analgesia, blood ingestion (as in endonasal pituitary surgery), or a sign of intracranial hypertension. Once these factors are excluded, pharmacological treatment can be initiated.

## 5. Neuroendoscopy and PONV

Postoperative nausea and vomiting (PONV) is a common and challenging complication in patients undergoing neuroendoscopic procedures. An incidence of up to 20% of cases has been reported in the literature [56].

Effective management of PONV in neuroendoscopy requires a multimodal approach that incorporates pharmacological interventions and procedural techniques. A recent trial showed that female gender and extended surgical approach were individual risk factors for postoperative nausea, and female gender, cavernous sinus dissection, and extended approach were individual risk factors for postoperative vomiting. While rates of PONV did not differ based on opioid dose, total operative time, smoking history, lesion size, surgical approach corridor, suprasellar involvement, presence of intraoperative leak, or use of lumbar drain, fat graft, or nasoseptal flap [7].

Pharmacological prophylaxis for PONV in neuroendoscopy, as well as in neurosurgery, commonly involves the administration of antiemetic agents prior to or during surgery.

While there is limited evidence on the optimal combination therapy for PONV, the use of a combination of ondansetron, scopolamine, and promethazine in patients undergoing endoscopic skull base surgery has been described. The authors conclude that the adoption of a multimodal prevention strategy should have several advantages; it should minimize the risk that moderate- to high-risk patients receive suboptimal prophylaxis, as well as minimize the risk of low-risk patients receiving single treatment that is not effective for the individual [7].

Aprepitant alone has also been recently studied in the neuroendoscopy setting. The authors noted that the preoperative use of aprepitant 80 mg reduced the incidence of postoperative vomiting and the use of postoperative antiemetics among patients undergoing TSA. In addition, although aprepitant did not reduce the absolute incidence of nausea, it did reduce the number of nausea episodes, potentially signifying the reduction of nausea severity [57].

In neuroendoscopic endonasal surgery, especially for pituitary tumors, maintaining an unobstructed surgical field and minimizing postoperative discomfort are crucial. One technique that aids in the prevention of PONV is the use of a reserved gastric tube during the procedure. This approach facilitates the decompression of gastric contents, thereby reducing the risk of postoperative vomiting. The tube is typically placed intraoperatively and left in situ during the immediate postoperative period [13]. Careful intraoperative management, including the avoidance of excessive irrigation fluid and limiting head manipulation, can also mitigate the risk of PONV. The use of total intravenous anesthesia (TIVA) with propofol instead of volatile agents has been shown to reduce PONV rates due to the absence of emetogenic inhalation anesthetics. Additionally, multimodal analgesia with minimal opioid use is recommended to lower the emetic burden.

An effective strategy for minimizing PONV in neuroendoscopic patients combines pharmacological prophylaxis with optimized anesthetic techniques and procedural adjustments. Utilizing a combination of ondansetron, dexamethasone, and, if necessary, metoclopramide ensures broad coverage of potential emetic pathways. Additionally, procedural considerations such as the reserved gastric tube and minimizing opioid use are fundamental in reducing the risk of PONV.

Given the potential for PONV to impact patient recovery and length of hospital stay, it is essential to adopt a proactive approach in neuroendoscopic practice. Further studies are warranted to establish standardized protocols that incorporate both pharmacological and procedural elements tailored to individual patient profiles.

## 6. Conclusions

Postoperative nausea and vomiting (PONV) remain a significant concern in neuroendoscopic procedures due to their potential impact on intracranial pressure, hemodynamic stability, and overall patient recovery. The incidence of PONV in neuroendoscopy appears to be influenced by multiple factors, including patient-related risk factors (e.g., female gender, younger age, history of motion sickness), anesthesia-related factors (e.g., opioid use, inhalational anesthetics), and procedure-specific characteristics (e.g., infratentorial approaches, cavernous sinus dissection).

While the endoscopic approach offers advantages such as reduced tissue trauma, shorter recovery times, and lower morbidity rates compared to traditional craniotomies, its proximity to brainstem structures and cerebrospinal fluid alterations may predispose patients to a higher risk of PONV. Current evidence supports a multimodal strategy for PONV prevention, incorporating pharmacologic interventions (e.g., 5-HT3 receptor antagonists, NK1 antagonists, dexamethasone, dopaminergic agents), anesthetic management (e.g., total intravenous anesthesia with propofol), and supportive measures (e.g., adequate hydration, oxygen therapy, scalp blocks, gastric tube).

Despite growing awareness of PONV in neuroendoscopic surgery, specific risk stratification models and targeted prophylactic protocols remain underdeveloped. Future research should focus on identifying procedure-specific predictors of PONV, optimizing perioperative management strategies, and evaluating novel antiemetic therapies in this patient population. Given the significant impact of PONV on neurosurgical outcomes, a standardized, evidence-based approach to prevention and treatment is essential to improving postoperative recovery and patient satisfaction in neuroendoscopic procedures.

## Figures and Tables

**Table 1:** Summary of Selected Studies on PONV in Neuroendoscopy.

Author(s)	Year	Study Type	Focus	Main Findings
Shim et al. [1]	2017	Narrative Review	Current and future perspectives on neuroendoscopy	Describes advantages and indications of neuroendoscopy
Chen et al. [8]	2020	Meta-analysis	Antiemetic drugs efficacy after craniotomy	Ramosetron and fosaprepitant most effective
Birkenbeuel et al. [7]	2022	Retrospective Study	PONV predictors after skull base surgery	Female gender, extended approach linked to PONV
Sato et al. [9]	2013	Retrospective Study	SIH and PONV after craniotomy	SIH linked to increased PONV
Gupta et al. [10]	2014	RCT	Granisetron vs. ondansetron for PONV	Both drugs reduce nausea; only droperidol reduces vomiting
Peng et al. [11]	2015	RCT	Dexmedetomidine for PONV reduction	Dexmedetomidine lowers PONV incidence
Akcil et al. [12]	2017	RCT	Scalp block vs. infiltration for pain and PONV	Scalp block lowers opioid need and PONV
Chen X et al. [13]	2024	Clinical Study	Gastric tube placement in endonasal surgery	Gastric tube lowers nausea incidence
Gupta A et al. [14]	2003	Systematic Review	Antiemetics and postdischarge nausea	Routine prophylaxis reduces postdischarge nausea
Pierre et al. [15]	2002	Observational Study	Apfel score and PONV prediction	Apfel score effective in PONV prediction
Eberhart et al. [6]	2002	Clinical Trial	Multimodal prophylaxis and satisfaction	Multimodal antiemetic strategy improves satisfaction
Gan et al. [16]	2020	Consensus Guidelines	Fourth guidelines on PONV management	Standardized PONV management recommendations

**Table 2:** Apfel Score.

Risk Factors	Points
Female gender	1
Nonsmoker	1
History of PONV	1
Postoperative opioids	1
Total	0–4

**Table 3:** Antiemetic Drugs for PONV Prophylaxis.

Drug	Class	Mechanism of Action	Recommended Dose	Common Side Effects
Ondansetron	5-HT3 antagonist	Blocks central and peripheral 5-HT3 receptors	4–8 mg IV	Headache, fatigue, QTc prolongation
Droperidol	D2 antagonist	Long-acting dopamine antagonist	0.625–1.25 mg IV	Sedation, dysphoria, QTc prolongation
Dexamethasone	Glucocorticoid	Prostaglandin antagonism and anti-inflammatory effect	4–8 mg IV	Hyperglycemia, delayed antiemetic effect
Metoclopramide	D2 antagonist	Blocks dopamine receptors and enhances GI motility	10 mg IV/IM/oral	Extrapyramidal symptoms, tardive dyskinesia
Aprepitant	NK1 antagonist	Blocks NK1 receptors (substance P)	40–80 mg oral	Headache, fatigue, hiccups
Scopolamine	Antimuscarinic	Competitive inhibition of muscarinic receptors	1.5 mg transdermal patch	Dry mouth, sedation, mydriasis
